# In the Right Place, at the Right Time: Autobiographical Case Report of an Ophthalmologist Who Had a Rhegmatogenous Retinal Detachment

**DOI:** 10.7759/cureus.21247

**Published:** 2022-01-14

**Authors:** Charisma Evangelista

**Affiliations:** 1 Ophthalmology, Wilford Hall Ambulatory Surgical Center, San Antonio, USA

**Keywords:** labor, labor and birth, labor and delivery, ophthalmologist, childbirth, rhegmatogenous retinal detachment, autobiographical case report

## Abstract

Rhegmatogenous retinal detachment (RRD) is a medical eye emergency that can lead to loss of vision, especially if not promptly treated. High myopia, retinal holes or tears, previous surgery, and trauma have been identified as risk factors for developing RRD. Although some obstetricians may believe labor or vaginal delivery increases the risk for RRD, there has been no significant evidence in the literature to support a correlation. This is a case report of a retinal detachment a month after childbirth as experienced by me, an ophthalmologist.

## Introduction

Putting oneself in patients’ shoes often helps in empathizing better and understanding what the patient is going through. However, it can be more enlightening when the doctor becomes the patient of their subspecialty. This case report aims to share my experience, as an ophthalmologist, of rhegmatogenous retinal detachment (RRD) a month after spontaneous vaginal delivery and my road to recovery.

## Case presentation

I was a 31-year-old female, an ophthalmology resident, with a history of high myopia. About a month into my maternity leave, I decided to return to my residency training to avoid a significant period of leave of absence and maintain my surgical skills as a senior resident. Just like any routine surgical day, I had several cataract surgeries lined up under the direct supervision of my attending. After the first case, I noticed a new floater in my dominant eye. I have had floaters in the past after undergoing laser retinopexy for lattice degeneration with subretinal fluid in medical school. However, this new floater looked different. It started inferiorly at the 5 o’clock position of my peripheral visual field. It looked like a dome of fluid similar to a teardrop accumulating in the outer corner of my eye. I kept trying to rub it off, but it was not going away. Instead, it became more noticeable each time I stepped out of the operating room as I walked the brightly lit hallways that led to the patient recovery area. My suspicion that it was a retinal detachment (RD) grew, but I was in denial since I saw well. I felt compelled to finish my surgeries before getting my eye checked.

I told our chief resident who happened to be in the retina clinic that day that I need a dilated eye examination. He agreed to examine my “floater.” Upon his confirmation, my heart sank in disbelief. It was a rhegmatogenous retinal detachment (RRD). I had work to finish, patients to call, and a baby and a toddler to tend. I kept going with my work as I waited for our retina staff’s expert medical advice.

Fortunately, there were three retina staff in-house. We discussed the treatment options as my RRD started to descend and rapidly involve the macula. I opted to undergo pneumatic retinopexy that same evening, which felt to be the most conservative treatment option and offered the fastest recovery. I went home that night and tried my best to comply with face-down positioning. We set up a table, chair, and multiple pillows to help me succeed with the positioning requirements. However, I kept waking up on my side. It was more difficult than I thought.

The next day, I had thousands of floaters severe enough to impede driving. Furthermore, the RD was still present, and my macula was not flat. So I underwent pars plana vitrectomy (PPV) with a scleral buckle (SB) placement. I knew developing a cataract was a significant risk, but I did not expect it to form intraoperatively and impede my surgeon’s view. They had to perform a lensectomy, which left me aphakic. Thankfully, I was under general anesthesia because the expected two- to three-hour surgery lasted for six hours instead.

The recovery period was complicated by acute angle-closure glaucoma (ACG) episode requiring a laser peripheral iridotomy (LPI), persistent inflammation requiring prolonged topical steroid use, steroid response ocular hypertension requiring several topical aqueous suppressants, medicamentosa-related follicular conjunctivitis, cystoid macular edema (CME), ptosis, and vertical strabismus.

About six months into my recovery, the majority of the complications resolved, except I was still aphakic and relied on a high plus soft contact lens. When it was time to implant an intraocular lens (IOL), it was tempting to aim for emmetropia. However, I would need laser vision correction in the contralateral eye to treat the resulting high anisometropia. Since I was not qualified for laser in situ keratomileusis (LASIK) and I was at high risk for steroid response-related ocular hypertension with photorefractive keratectomy (PRK), one of my mentors advised me to simplify my goals and aim for the least amount of surgeries and potential complications. It was the wisest advice because, after my IOL implantation, I simply updated my eyeglasses and have not required any further surgeries since.

Despite still wearing high myopic spectacles, being independent of eyedrop and seeing 20/20 is a huge success. Since my recovery from my RRD repair and secondary IOL implantation, I was cleared by multiple retina specialists to return to duty. Since then, I have safely performed several hundreds of surgeries and seen thousands of patients in a large academic center under regular peer review. Over five years later, I was able to participate in humanitarian missions, complete my cornea and refractive surgery fellowship, and serve as an expeditionary ophthalmologist in a combat zone.

## Discussion

The rate of patients regaining a visual acuity of 20/20 after macula-off (detached macula) RD is difficult to ascertain due to the scarcity of reported data. One study reported that about 40%-80% of patients with macula-off RD repair have a postoperative vision of 20/50 or better. The predictors of visual recovery include preoperative visual acuity, young age, the height of macular detachment, and a shorter duration of detachment [1].

Young age may have contributed to my excellent visual outcome, but the fact that I had a macula-off RRD placed me at an increased risk of not having a postoperative vision of 20/20. The immediate diagnosis and surgical treatment, which minimized the duration of detachment, likely increased the chance of success in my case.

Retinal detachment and childbirth

Is there an association between RD and childbirth? High myopia and previous history of lattice degeneration with subretinal fluid put me at high risk for an RD. I had a dilated eye examination during my second trimester, and no new retinal tears or detachments were seen. However, could active and aggressive pushing and Valsalva during labor lead to an RD?

Prior survey studies reported that some obstetricians advocate for assisted delivery via cesarean section or instrumental delivery instead of spontaneous vaginal delivery for patients who have a history of RRD repair [2,3]. However, several studies in the literature suggest that no correlation exists between RD and labor or spontaneous vaginal delivery [4-7].

Lessons learned from a surgeon’s perspective

Intraoperative and postoperative complications, no matter how statistically low, should always be considered and planned for. The incidence of intraoperative cataract formation during PPV is not well documented in the literature. The incidence of lens touch during PPV has been reported to vary from 0.9% to 3.7% [8,9]. In my case, the cataract formed during the early part of the PPV, and my surgeons had to perform an emergent unplanned lensectomy in order to complete the rest of the RRD repair.

One meta-analysis reported elevated intraocular pressure as an early postoperative complication in 5.4% after SB placement and 11.6% after PPV [10]. This study also reported diplopia/extraocular muscle dysfunction as a late postoperative complication in 2.7% after SB placement and 0.5% after PPV. In addition, this study reported the rate of CME to be about 0.03% after both SB placement and PPV. All of these complications occurred in my eye postoperatively, which highlights the importance of proper informed consent to help better prepare the patient especially when complications arise.

There is about a 1.4%-4.4% incidence of acute ACG after an SB procedure and about 6%-18% incidence of increased intraocular pressure after intravitreal gas injection depending on the type and amount of gas injected [11]. ACG secondary to a pupillary block after a combination of SB, PPV, and gas tamponade in an aphakic eye has been previously reported [12]. As mentioned above, I was not aphakic preoperatively but had to undergo an emergent lensectomy intraoperatively, in addition to the SB, PPV, and gas tamponade. The combination of these procedures likely led to the mechanical narrowing of my iridocorneal angle, which increased the risk for acute ACG postoperatively, especially when I took breaks from my face-down positioning. A prophylactic surgical iridotomy intraoperatively may help prevent this complication. However, a good discussion of strict return precautions (such as acute onset headache, eye pain, halos, nausea, or vomiting) with the patient is still warranted in case the iridotomy fails.

Broadening the differential and/or seeking another expert’s opinion may help find solutions to a chronic or recurrent condition. With multiple procedures and complications, I had to be on multiple eye drops for over a year, including topical steroid and antiglaucoma eye drops. I developed chronic eye redness, photophobia, and recurrent mild anterior chamber reaction, which was initially thought to be due to rebound uveitis by multiple subspecialists. I was finally referred to another uveitis specialist from another institution who diagnosed me with medicamentosa-induced chronic follicular conjunctivitis. They recommended tapering the steroid eye drops, discontinuing my current glaucoma drops, starting with just one preservative-free antiglaucoma medication, and then monitoring my ocular response. Using preservative-free glaucoma medications has been shown to significantly improve ocular signs and symptoms, including conjunctival redness, conjunctival follicles, and blepharitis [13]. My surgeons concurred with the plan, and eventually, my chronic condition resolved.

When it comes to visual rehabilitation, being a patient and not rushing into surgery are important lessons in this case. Allowing the eye to heal eventually led to the resolution of the ptosis and vertical diplopia. Allowing the eye to fully recover, albeit slowly, avoided multiple surgeries and possible complications associated with each intervention.

As a member of the teaching faculty, I share this experience with residents when discussing pertinent cases such as operating on a high myope with a unilateral cataract who may be at a higher risk of RD or postoperative anisometropia. This journey also taught me patience and humility. It involved the acceptance of the possibility that I may not be able to get rid of my glasses, even as a refractive surgeon. I am grateful that I can still perform surgeries and help others achieve their refractive goals.

Even though I am an anterior segment surgeon, every patient encounter to this day brings me back to my journey. Whether it is for an elective surgery or an acute visit for a decrease in vision, I am now more attentive and sensitive to patient concerns. I often share my insight, not only as a surgeon but as a prior patient.

Lessons learned from a patient’s perspective

When there is a superior RD, fluid under the retina may shift or flow downward with gravity while the patient is upright. As the fluid shifts, it can travel across the macula and evolve from a macula-on RD (attached macula) to a macula-off RD. Macula-off RD may have a worse prognosis than a macula-on RD since it already involves the macula, which is responsible for central vision.

As mentioned above, after I knew I had an RD, I still kept working and did not rest or lay down while waiting for my doctor. This likely hastened the shifting of the subretinal fluid into my macula, and I saw my central vision worsen while I was finishing my work. With this experience, I have been recommending activity restrictions to patients with RD while they are waiting for their retina referral in order to decrease the chance of fluid shift, especially if it has not involved the macula yet.

Face-down positioning requirement after RD repair is necessary either after a PPV with gas tamponade or after a pneumatic retinopexy to reattach the retina. I had to do face-down positioning postoperatively after both procedures. After the pneumatic retinopexy, I was discharged home and instructed to do face-down positioning overnight. I tried using pillows, chairs, and a table to help keep my face-down position. However, I kept waking up either sideways or supine. A commercial face-down pillow or face-down recovery equipment may have increased my success, but I did not have access to those at that time. After my SB/PPV/gas tamponade surgery, I was admitted overnight, and the nursing staff was able to help me maintain my positioning requirements.

Finally, as an RD patient, understanding and expecting multiple surgeries and potential complications may help both the patient and the family face the potential challenges ahead. For instance, surgery or recovery may take longer than expected, more unplanned surgeries may happen, return to school or work may be delayed, and more help at home may be needed for a significant period of time. Setting these expectations and planning ahead of time may help pave the way for a smoother road to recovery.

## Conclusions

This experience highlights the fact that being an ophthalmologist while having a retinal detachment helped me recognize the urgency of this condition. However, it does not make the acceptance of the condition and choosing the treatment options easier. This experience has taught me life lessons that continuously help me in clinical practice. It has taught me humility and gave me a unique patient perspective, which often inspires me to make sure patients will also feel that they are in the right place, at the right time.

## References

[1] Ross WH (2002). Visual recovery after macula-off retinal detachment. Eye (Lond).

[2] Papamichael E, Aylward GW, Regan L (2011). Obstetric opinions regarding the method of delivery in women that have had surgery for retinal detachment. JRSM Short Rep.

[3] Elsherbiny SM, Benson MT (2003). Retinal detachment and the second stage of labour: a survey of regional practice and literature review. J Obstet Gynaecol.

[4] Ellabban AA, Patil AD, Yorston D (2020). Risk of retinal detachment during labour: beliefs versus evidence. Obstet Gynecol.

[5] Gomez J, Rice SM, Makhamreh MM, Al-Kouatly HB (2020). Pregnancy management in a patient with stickler syndrome. Mol Genet Genomic Med.

[6] Landau D, Seelenfreund MH, Tadmor O, Silverstone BZ, Diamant Y (1995). The effect of normal childbirth on eyes with abnormalities predisposing to rhegmatogenous retinal detachment. Graefes Arch Clin Exp Ophthalmol.

[7] Patil AD, Ellabban AA, Patil DB (2020). Ocular manifestations of pregnancy and labour: from the innocuous to the sight threatening. Obstet Gynecol.

[8] Jackson TL, Donachie PH, Sparrow JM, Johnston RL (2013). United Kingdom National Ophthalmology Database Study of Vitreoretinal Surgery: report 1; case mix, complications, and cataract. Eye (Lond).

[9] Elhousseini Z, Lee E, Williamson TH (2016). Incidence of lens touch during pars plana vitrectomy and outcomes from subsequent cataract surgery. Retina.

[10] Lv Z, Li Y, Wu Y, Qu Y (2015). Surgical complications of primary rhegmatogenous retinal detachment: a meta-analysis. PLoS One.

[11] Sahoo NK, Balijepalli P, Singh SR, Jhingan M, Senthil S, Chhablani J (2018). Retina and glaucoma: surgical complications. Int J Retina Vitreous.

[12] Kumar A, Kedar S, Garodia VK, Singh RP (2002). Angle closure glaucoma following pupillary block in an aphakic perfluoropropane gas-filled eye. Indian J Ophthalmol.

[13] Pisella PJ, Pouliquen P, Baudouin C (2002). Prevalence of ocular symptoms and signs with preserved and preservative free glaucoma medication. Br J Ophthalmol.

